# Displacement and Mental Health Problems in Children and Adolescents of a Colombian Indigenous Population

**DOI:** 10.3389/ijph.2025.1607782

**Published:** 2025-08-26

**Authors:** Felipe Agudelo-Hernández, Laura Inés Plata-Casas, Eduardo Marulanda-López

**Affiliations:** ^1^ Universidad de Manizales, Facultad de Ciencias para la Salud, Manizales, Caldas, Colombia; ^2^ Ministerio de Salud y Protección Social, Mental Health, Bogotá, Colombia

**Keywords:** suicide, child behavior, Indigenous peoples, food security, armed conflicts

## Abstract

**Objectives:**

To explore the relationship between forced displacement and variables associated with mental health in an indigenous Colombian pediatric population.

**Methods:**

A mixed methods design was applied with 69 children aged six to 16 years belonging to 45 families of the Embera Dobidá People: 25 displaced families and 20 non-displaced families. The qualitative phase involved focus groups, while the quantitative phase used a cross-sectional correlational design.

**Results:**

Quantitative findings revealed statistically significant correlations (p < 0.001) between displacement and multiple mental health indicators, particularly emotional and behavioral problems. Food insecurity showed a moderate but meaningful effect, highlighting the impact of nutritional vulnerability on the emotional wellbeing of displaced children and adolescents. In the qualitative phase distinct themes emerged in each community: *A territory that cannot be returned* to, in Caldas, and *The angry and dying territory*, in Chocó.

**Conclusion:**

For Indigenous communities in Colombia, mental health is closely tied nutritional sufficiency and a meaningful relationship with ancestral territory. These elements are disrupted by forced displacement, contributing to elevated suicidal risk and emotional distress among children and adolescents.

## Introduction

Suicide is a particularly relevant phenomenon in Indigenous populations, with rates consistently higher than in other non- Indigenous youth populations [1–4]. Although previous attempts constitute the main risk factor, in Indigenous children and adolescents the causes have been linked to structural inequalities, historical marginalization, loss of cultural identity and fractured community fabric [1–3]. Global studies have documented that suicide in Indigenous communities is associated with social inequity, barriers in access to health services and persistent cultural vulnerabilities [5, 6].

In Colombia, the suicide rate per 100,000 population ranged between 4 and 5.7 between 2010 and 2019, while in children and adolescents aged 5–19 years it varied between 2.6 and 3.5 [7]. In Indigenous populations, a worrying increase has been identified: from 63 deaths by suicide in 2017 to 98 in 2020 [2]. Regions such as Amazonas and Vaupés have reported the highest rates in the country within these communities [7].

The link with the land constitutes a central axis in the identity and wellbeing of Indigenous peoples. For the Embera Dobidá community, the land not only represents a physical space, but also a spiritual one, from which emanate the natural elements that provide healing, sustenance and balance [8, 9]. Forced displacement has fractured this relationship, intensifying processes of westernization, acculturation and economic dependence, which has generated cultural stress, frustration and spiritual disharmony [9, 10].

In the case of the Embera Dobidá, a population of approximately 16,000 people inhabiting the Chocó rainforest, the suicide rate reached 247.9 per 100,000 inhabitants in 2019, representing 0.87% of the population, with 50% of the cases occurring among young people and adolescents [2, 11]. A decade ago, no suicides were reported in this community [11].

These factors are compounded by new interconnected threats that make up what has been called a polycrisis: a intersection of simultaneous crises - environmental, economic, social and cultural - that disproportionately affect the most vulnerable populations, such as Indigenous communities. Symptoms in the pediatric population act as an early warning system of a society in crisis, manifesting the cumulative effects of threats such as climate change, economic insecurity, political polarization and loss of social cohesion [12].

Within these critical factors, solastalgia has emerged as a key concept to understand the emotional suffering derived from the transformation or loss of the natural environment. In Indigenous communities, this distress is intensified when the ancestral territory is degraded or when people are displaced from it, affecting their sense of belonging, identity and spiritual wellbeing [13]. In addition, it has been documented that the increase in temperature and extreme weather events affect sleep patterns, mood, anxiety, depression and other mental disorders, while increasing exposure to violence and substance abuse [14–16].

Another factor that adds to the concept of polycrisis [12] is food insecurity, which is associated with malnutrition and deficiencies of micronutrients essential for neurological function and mental health [17]. This influences cognitive development and the risk of mental health problems in childhood that require cultural adaptive approaches in crisis contexts, improved accessibility and follow-up, given the risk of the triple burden of malnutrition: undernutrition, hidden hunger and obesity [17–20].

### The Present Study

Research on Indigenous pediatric mental health remains limited. Therefore, this study aims to explore in a Colombian Indigenous population that experienced forced displacement, the relationship between variables associated with mental health, including general health, parenting, behavior, emotions and suicidal risk, with other variables such as food security, climate anxiety and relationship with the territory. We also sought to compare these variables with a similar population without forced displacement. The hypothesis states that there is a significant relationship between pediatric mental health, food security, general health, climate anxiety and connection with the territory, with displacement and living conditions being determining factors in the wellbeing of this population.

## Methods

This mixed study was conducted during the first semester of 2023 with a design that included a qualitative and a quantitative phase. In the qualitative phase, focus groups were conducted with children, youth and their caregivers, followed by a thematic analysis. In the quantitative phase, a cross-sectional and correlational study was conducted to explore the associations between mental health, food security, climate anxiety and other factors related to the wellbeing of the pediatric population that included children and adolescents belonging to Emberá Dobidá communities located in the departments (states) of Caldas and Chocó, Colombia.

### Participants

This study was conducted with two populations: The children, adolescents and caregivers of the Emberá Community, displaced by the armed conflict, in Caldas, and the children and adolescents of a Community in Chocó, which shares geographic proximity of origin and customs with the displaced community. According to the National Population and Housing Census of the National Statistics Department, the Embera people -which includes the Chami, Katío, Dobidá and Wounan subgroups-has a population of 56,504 people. In 2019, 119 people from the Embera Dobidá people (Dobidá means “people of the river”) of Alto Baudó, Chocó, were displaced due to violence.

It is important to note that these people were victims of the conflict and not actors of violence. The territory where they arrived has a polluted river with no fish, lacking access to drinking water. In 2024, the community had 158 inhabitants, of which 17.74% were children under 5 years of age and 25.3% belonged to the six to 16 age group, constituting the entire population participating in this study.

### Data Collection and Study Design

The qualitative phase and the quantitative phase were designed as complementary studies.

### Qualitative Phase

The children and adolescents of the Emberá community of Caldas belonged to 25 families. Of these families, five participated in the focus groups for the qualitative phase of the study, for a total of 14 people in this group. The children and adolescents of the Chocó community belonged to 20 families, of which four participated in the focus groups, for a total of 12 people in this group.

For the convening of the focus groups, the Secretariat of Health of Chocó and Caldas were involved, within the framework of public mental health projects. The Chocó group was selected according to its proximity, both culturally and geographically, to the Caldas community. The selection of participants was according to age and sex of the initial community. The group selected from the displaced community corresponds to 100% of the children and adolescents in the community. The group from Chocó corresponds to 70% of that community.

Inclusion criteria required participants to be members of the communities, to be over 6 years of age and, in the case of those under 18 years of age, to have a guardian participating in the group. No exclusion criteria were applied.

For the development of the group, a clear objective was established for each community and participants were selected based on their willingness to participate. Moderators and translators from the same communities facilitated the sessions and participated. Informed consent was obtained from adult participants and consent from minors before the sessions began. Minutes of the meetings were compiled and validated with community leaders and those participants who were able to attend a follow-up meeting.

The groups were led by a psychiatrist, close to both communities due to previous ethnographic processes, with a doctorate in social sciences, childhood and youth, as well as intercultural leaders from Chocó and Caldas. To guide the conversation, questions related to perceived changes in the territory, meanings about displacement and the relationship between place of origin and current wellbeing were incorporated into the interview protocol.

The translation process was carefully managed: interviews and interactions were conducted in the participants’ mother tongues with the assistance of an ethno-educator, a native speaker and member of the community. The translation process was iterative and participatory, incorporating back-translation and community validation to preserve meaning and avoid loss of nuance.

The recordings were initially conducted in Embera (a spoken but not written language) and subsequently translated into Spanish for analysis, thus ensuring the accuracy of the community’s psychological and cultural responses to environmental changes. The data were analyzed using thematic analysis, according to the checklist based on the consolidated COREQ criteria [21]. This approach involved initial coding of the data and subsequent identification of themes that would accurately capture participants’ experiences and perspectives. N-VIVO software was used to perform open, axial and selective coding, which facilitated the systematic construction of the main thematic categories. In addition, the interpretations were validated through participant verification, with the support of a translator, which reinforced the credibility of the findings. This method allowed a nuanced identification of recurrent themes related to the research topics, ensuring that the analysis process was clearly documented and reproducible [22].

### Quantitative Phase

Validated instruments were applied to the 25 families of the Caldas community and to the 20 families of the Chocó community to evaluate variables of mental health, food security and physical wellbeing.

### Instruments

The sociodemographic survey included questions on age, gender, family composition, reasons for migration and difficulties in the place of residence. Responses to the latter were categorized.

#### PACES Scale (Parenting, Behavior, Emotions, and Suicide Risk)

Evaluated mental health problems and suicidal risk in children and adolescents. It consists of 42 items divided into two parts: one addressed to parents and the other addressed to children and adolescents aged six to 16 years. A Likert format was used to measure frequency and severity of symptoms. This scale was previously validated in Indigenous communities and showed adequate measures of reliability and structural configuration. The instrument demonstrated high reliability, with a Cronbach’s alpha of 0.911, developed in the study community [23].

#### Colombian Household Food Security Scale (CHFSS)

Instrument of rapid application with high validity and reliability in similar contexts. For validation in Colombia, the scale was proposed with variables such as food insecurity without hunger and food insecurity with hunger, with a Cronbach’s alpha greater than 0.89. [24]. It was specifically validated in the Embera population and translated into the Embera language for its correct application by the ethnoeducator.

#### Solastalgia Scale

Psychometric instrument designed to assess the emotional distress associated with the transformation or loss of the natural environment, especially relevant in Indigenous communities affected by forced displacement or environmental degradation. In this study, a brief version validated in Colombia was used, composed of five items that measure feelings such as sadness, helplessness and disconnection with the territory, using a Likert-type scale.

This tool showed high internal consistency (α > 0.85), unidimensionality and convergent validity with indicators of mental health and connection with nature [25].

#### Pediatric Evaluation

Anthropometric variables, signs of malnutrition, anemia, respiratory symptoms, gastrointestinal symptoms, oral health, skin health, neurodevelopmental aspects, signs of maltreatment and other general symptoms were considered. It was grouped in a matrix with dichotomous variables, summarizing the findings as “Pediatric health problems”. These variables were determined by pediatricians who were part of the research team.

### Analysis

The quantitative analysis of the study was carried out using SPSS, where means, standard deviations and proportions were calculated, in addition to applying bivariate correlations to evaluate the relationships between mental health and environmental variables. When the normality of the variables was verified, the difference between means was determined by means of statistical tests such as Student’s t-test and the effect size was calculated with Cohen’s d.

For the qualitative phase, we used thematic analysis based on Braun & Clarke’s approach [22], with the support of NVivo software, used exclusively for the coding and organization of the qualitative data obtained in the interviews. On the other hand, Stella Architect Ink version 22 [26] was used to model systemic dynamics and visualize emerging patterns in the relationship between territory and community wellbeing, integrating qualitative and quantitative results in a comprehensive analysis framework.

## Results

The study included 69 children and adolescents, with a mean age of 9.23 years (SD = 2.41). Regarding gender, 53.7% were girls (n = 37) and 46.3% were boys (n = 32). Regarding family structure, 75% (n = 52) of the participants lived with father, mother and siblings, while 25% (n = 17) consisted only of mother and children, because the parents were killed in Alto Baudó, Chocó. 62.5% (n = 43) reported not having had a job in the last 3 months, reflecting a precarious economic situation.

All the participants indicated that their main occupation was agriculture or fishing, which is evidence of their dependence on natural resources for their subsistence. In the pediatric evaluations, relevant health conditions were identified in the population under 16 years of age: dental caries in 35% (n = 24) of the children, palmar pallor in 30% (n = 21), scabies in 30% (n = 21), fever in 30% (n = 21), chronic malnutrition 50% (n = 35, CI95%: 40.1%–59.9%), incomplete vaccination schedule in 20% (n = 14).

### Quantitative Phase

In the results obtained through the PACES Scale, it was identified in the displaced Indigenous community that 30% (n = 21, CI95%: 21.7%–38.3%) of the children and adolescents presented suicidal risk. In addition, 50% (n = 35, 95% CI: 40.6%–59.4%) showed emotional problems, 20% (n = 14, 95% CI: 12.3%–27.7%) showed behavioral problems, and a similar percentage showed parenting problems.

On the other hand, 95% (n = 66) of the caregivers stated that they would not return to their territory of origin, despite the difficulties they face in their new environment. Table 1 shows other descriptive data for the two study communities, with a higher score for mental health problems in the community that experienced forced displacement and a higher score on the Solastalgia scale for the community that remains in Chocó.

**TABLE 1:** Sociodemographic and descriptive data of the instruments, Caldas, Colombia, 2024-2025.

Variables	Community	Mean	Deviation	Avg. error
Age	Embera Caldas	14.97	3.017	0.366
	Embera Chocó	14.32	2.634	0.319
Parental Involvement	Embera Caldas	1.93	1.633	0.198
	Embera Chocó	1.63	1.132	0.137
Parental Adjustment	Embera Caldas	2.32	1.832	0.222
	Embera Chocó	2.41	1.677	0.203
Suicide Risk	Embera Caldas	3.35	2.472	0.298
	Embera Chocó	2.68	2.161	0.262
Behavioral problems	Embera Caldas	2.46	1.731	0.21
	Embera Chocó	1.97	1.349	0.164
Emotional problems	Embera Caldas	5.51	2.127	0.258
	Embera Chocó	4.38	1.657	0.201
Total PACES	Embera Caldas	15.39	6.107	0.735
	Embera Chocó	13.07	4.5	0.546
Solastalgia	Embera Caldas	10.71	2.961	0.356
	Embera Chocó	13.88	5.042	0.611
Body mass index (BMI/Z)	Embera Caldas	0.43	1.331	0.161
	Embera Chocó	0.87	0.96	0.116
Total medical findings	Embera Caldas	3.16	2.19	0.266
	Embera Chocó	1.53	1.298	0.157
Food Security Sum (FAO)	Embera Caldas	14.88	4.224	0.508
	Embera Chocó	12.94	3.314	0.402

Regarding food security in the displaced community, the results show a marked precariousness in access to essential resources. It was identified that 97.5% of the households reported lack of food, while 72.5% (n = 50) of the families expressed not having enough money to buy food for their children. This situation translates into severe food restrictions, with 77.5% (n = 54) of children going to bed hungry sometimes and 5% (n = 3) going to bed hungry every night.

In addition, 25% (n = 17) of the children consumed less than three meals a day, which increased the risk of malnutrition and affected their physical and cognitive development. Food shortages impacted unevenly by age, with seven- and eight-year-olds being the most affected by reduced food availability.

Statistically significant correlations (<0.001) were found between migration and emotional problems, solastalgia, body mass index, total medical findings and food security. Similarly, statistically significant correlations (<0.001) were found between food security and migration, suicidal behavior and other mental health problems (Total PACES) (Table 2).

**TABLE 2:** Correlations between mental health problems and other psychosocial factors, Caldas, Colombia, 2024-2025.

Variables	Migration	Parental involvement	Adjustment	Suicide	Behavioral problems	Emotional problems	Total PACES	Solastalgia	BMI/Z	Total medical findings	Food insecurity
Age	−0.114	0.017	0.086	−0.365**	−0.253**	−0.169*	−0.263**	−0.14	0.078	−0.277**	−0.043
Sig. (bilateral)	0.185	0.84	0.32	<0.001	0.003	0.049	0.002	0.103	0.369	0.001	0.623
Migration		−0.105	0.025	−0.144	−0.156	−0.287**	−0.212*	0.361**	0.188*	−0.416**	−0.249**
Sig. (bilateral)		0.224	0.77	0.093	0.071	0.001	0.013	<0.001	0.028	<0.001	0.003
Parental involvement			0.051	0.093	0.230**	0.301**	0.499**	0.149	0.011	0.062	0.129
Sig. (bilateral)			0.554	0.283	0.007	<0.001	<0.001	0.083	0.901	0.471	0.134
Parental adjustment				−0.125	0.052	−0.044	0.285**	0.087	0.074	−0.052	−0.068
Sig. (bilateral)				0.148	0.546	0.614	0.001	0.316	0.39	0.547	0.431
Suicide					0.412**	0.229**	0.630**	0.250**	0.032	0.558**	0.350**
Sig. (bilateral)					<0.001	0.007	<0.001	0.003	0.714	<0.001	<0.001
Behavioral problems						0.672**	0.798**	0.108	0.174*	0.413**	0.240**
Sig. (bilateral)						<0.001	<0.001	0.212	0.043	<0.001	0.005
Emotional problems							0.731**	0.049	0.103	0.401**	0.304**
Sig. (bilateral)							<0.001	0.571	0.232	<0.001	<0.001
Total PACES								0.222**	0.13	0.511**	0.384**
Sig. (bilateral)								0.009	0.132	<0.001	<0.001
Solastalgia									0.03	0.024	−0.016
Sig. (bilateral)									0.733	0.78	0.855
BMI/Z										−0.155	0.258**
Sig. (bilateral)										0.072	0.002
Total medical findings											390**
Sig. (bilateral)											<0.001

**The correlation is significant at the 0.01 level (bilateral). *Correlation is significant at the 0.05 level (bilateral). N = 137. BMI: body mass index.

When comparing both communities in relation to displacement, a moderate effect size and statistically significant associations were found for the variables emotional problems, parental adjustment, solastalgia, food security and migration. The effect sizes of the total medical findings are large (Table 3).

**TABLE 3:** Difference in means between the two communities, Caldas, Colombia, 2024-2025.

Variable	F	Sig.	Sig. (bilateral)	Difference in averages	SD	Effect size (d)
Age	3.693	0.057	0.185	0.647	2.84	0.22
			0.185	0.647		
Parental Involvement	4.746	0.031	0.224	0.294	1.407	0.21
			0.225	0.294		
Parental Adjustment	1.255	0.265	0.77	−0.088	1.75	0.05
			0.77	−0.088		
Suicide Risk	4.658	0.033	0.093	0.671	2.339	0.29
			0.093	0.671		
Behavioral problems	6.602	0.011	0.071	0.485	1.565	0.31
			0.071	0.485		
Emotional problems	7.05	0.009	0.001	1.132	1.983	0.57
			0.001	1.132		
Total PACES	6.582	0.011	0.013	2.318	5.475	0.02
			0.013	2.318		
Solastalgia	35.003	0.001	0.001	−3.172	4.409	0.72
			0.001	−3.172		
Body mass index (BMI/Z)	22.491	0.001	0.028	−0.441	1.972	0.01
			0.028	−0.441		
Total medical findings	15.331	0.001	0.001	1.632	1.177	1.39
			0.001	1.632		
Food Security (FAO)	0.593	0.443	0.001	1.943	3.909	0.49
			0.001	1.943		

### Qualitative Phase

The focus group participants are specified in Table 4 with some representative words mentioned during the groups. The thematic analysis showed different themes for each community. In the Embera Community of Caldas the theme was “A territory to which one cannot return”, made up of categories such as “Nostalgia for the place of origin” and “Having to earn more money”. In the Chocó Community the theme was “The Territory angry and dying”, made up of categories such as “Changes in the rain and sun” and “Contamination by the war”.

**TABLE 4:** Qualitative phase participants, Caldas, Colombia, 2024-2025.

Family	Age (years)	Sex	Representative words
Embera Dobidá Community of Caldas			
Family 1	16, 43	F, F	Although they miss the territory of origin, they know they cannot return because of the war
Family 2	32, 7, 9	F, M, F	Concern about food, because everything in the new place must be bought and not taken from the land
Family 3	7, 23, 10	M, F, F	It is difficult to play; there are few spaces to play. Missing the river of childhood
Family 4	29, 9	M, M	The desire not to live in the community, wanting to move to the city for a better future
Family 5	49, 9, 18, 6	F, M, M, M	Food must be bought, and there is no money or work. In the new place, only plantains can be planted
Embera Dobidá community of Chocó			
Family 1	69, 45, 7	M, F, M	Fish with a dead taste. Dead people coming down the river. Not being able to play in the river
Family 2	38, 14, 7	M, F, M	The sun stronger, the rain stronger. The sun beats down because the territory is angry
Family 3	36, 13, 8	F, F, M	Fear of conscription for war. Wanting to leave but unable to do so
Family 4	19, 9, 32	F, M, M	Grief for the dead of the community community. A sick and sad territory

In the Embera Dobidá Community of Caldas the theme was “A territory to which one cannot return”, made up of categories such as “Nostalgia for the place of origin” and “Having to earn more money”. In the Chocó Community the theme was “The Territory angry and dying”, made up of categories such as “Changes in rain and sun” and “Contamination by war.”

### A Territory to Which One Cannot Return: Embera Dobidá Community of Caldas

In this topic, the discomfort of not being able to return to their territory of origin in Chocó is noted. Among the representative quotes, the following is highlighted by the community’s ethno-educator: “We know that it is still there [in the place of origin], that the river is still there, but very dangerous. Even if we are enduring hunger here, we must stay.” [R_09]. To this, a young man from the community responded: “I remember how it was there. You could play more. I see my brothers here and it makes me sad because they can’t play like we played there”. [H_02]. Also, a woman from the community mentioned:

We are not one to remember much. We are calm with those who died. But one feels in a place where one is obliged to be. You feel like going back to a place that exists, but to which you cannot return. That does make you remember and miss a lot.

In response to this, a young woman from the community said: “Although the family who stayed there have told us that it is not like it used to be, that they are having a hard time there too”. [Y_04]

### The Territory Angry and Dying: Embera Dobidá Community of Chocó

In the theme that emerged in the community of Chocó, the impact of phenomena such as extractivism and the armed conflict on their wellbeing and relationship with the territory was pointed out. The community leader noted, “The fish taste bad, like death, the territory is sick and angry because of the death, the death of the mines to the trees and rivers, the death of the war to the men” [J_01].

An older woman from the community also said: “20 years ago suicide did not exist for us, that is new, before we did not even think about it. Of course, it is because now the earth is sick and angry… and sad.” [Ro_03]. Other adults and youth also mentioned perceptions on the weather, they pointed out aspects such as: “The sun now mistreats us” [D_05], “Crops are being damaged, we no longer understand the rain” [M_02], or “Nobody understands the weather now, the weather went crazy.” [S_01].

Another important aspect related to food security in this group was the impossibility of feeding themselves adequately due to the contamination of the rivers. Regarding this point, the community leader pointed out: “Where the dead go down, the fish go down. We cannot eat fish that feed on dead people who could even be family.” [J_03].

### Methodological Integration

**FIGURE 1:**
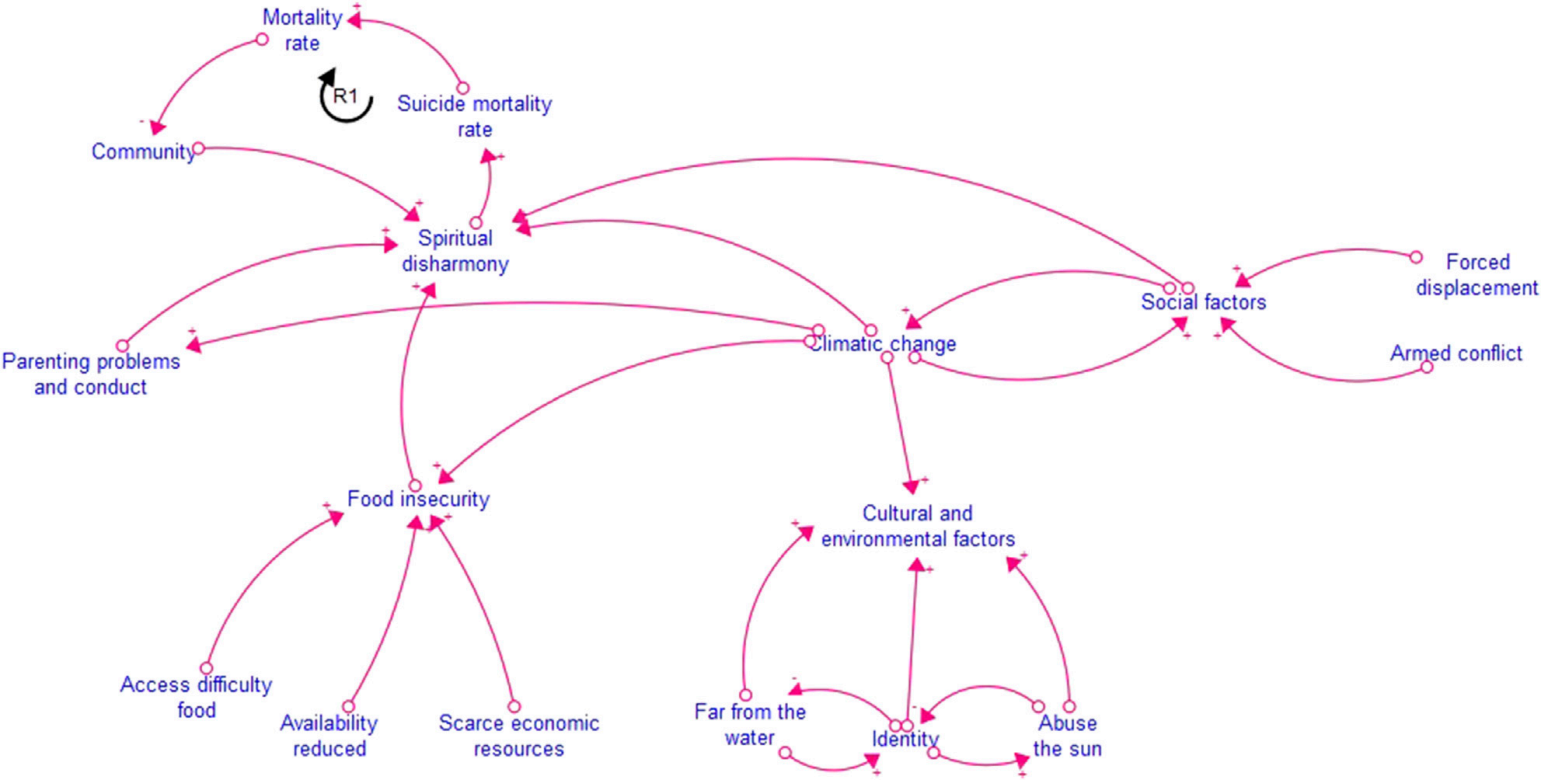
Hypothetical model of factors related to mental health problems in study communities (Caldas, Colombia, 2024-2025).

The hypothetical diagram of factors related to mental health problems (Figure 1) illustrates the interaction between spiritual disharmony, suicide mortality, food insecurity, displacement and environmental impact in the Indigenous communities studied. Loop R1 shows how spiritual disharmony - a result of territorial insecurity, loss of identity and social precariousness - reinforces the suicide mortality cycle, weakening community resilience and collective approaches to prevention.

The model clarifies that while social factors do not directly modify climate change, they do modulate its impact on the community. Social and cultural insecurity aggravates the effects of climate change by limiting adaptive capacity, reducing access to natural resources, food security and environmental mitigation strategies.

## Discussion

This study explored the relationship between variables associated with mental health and food security of two Embera Dobidá Indigenous Peoples in Colombia, one that experienced migration due to the armed conflict and the other without this factor, emphasizing the impact of migration on these variables. The hypothesis was confirmed by determining the relationship between pediatric mental health, food security, general health, solastalgia and forced displacement. This last factor especially influenced food security and non-psychiatric medical conditions.

In some populations, suicide rates in children and adolescents are 30 times higher than in the general population, and it has been shown that food insecurity and migration are linked to mental disorders and a withdrawn attitude [2, 16]. These findings support the hypothesis that in Indigenous communities, food crises not only impact nutrition, but also affect emotional regulation, which may increase the risk of suicidal behavior.

In this sense, the results also showed that migration could impact suicidal behavior, without impacting solastalgia, although a statistically significant correlation between solastalgia and suicide in these communities is found in the correlations. This could indicate that those who persist in the territory might have more distress about perceived changes in their territory.

Qualitative and quantitative findings show that participants face a polycrisis, understood as the simultaneous convergence of multiple interrelated and mutually reinforcing crises. In this case, it is a unique combination of environmental, economic, social and cultural threats, including structural poverty, food insecurity, natural resource dependence, vulnerability to climate change and forced displacement. This overlap of critical factors not only increases exposure to risk but also limits the community’s capacity to respond and adapt.

In addition, the lack of state recognition and the weakening of traditional community structures intensify the sense of isolation, uprooting and identity crisis. In this context, the polycrisis not only affects material living conditions, but also erodes symbolic and spiritual ties with the territory, which are fundamental for psychosocial wellbeing in Indigenous communities [1–6, 25].

The participants’ narratives reinforce these findings. In Caldas, the community expressed nostalgia for the lost territory and the impossibility of returning due to the armed conflict. In Chocó, there was evidence of a perception of “sick territory”, affected by extractivism and war, which has transformed their relationship with nature and generated feelings of sadness, anger and disconnection.

Several studies have shown that family and social factors play a determining role in the wellbeing of children. In Indigenous communities, family support and exposure to adverse childhood events may influence the development of affective problems and vulnerability to suicidal behavior [1, 2, 9, 11, 15]. These findings are consistent with the present investigation, where it was observed that suicidal risk correlates significantly with loss of territory and with the perception of being in an alien culture. These data coincide with research in humanitarian crisis contexts, such as in Lebanon [27] and Israel [28], where food insecurity has been associated with emotional deterioration in displaced children, also mediated by environmental and psychosocial factors, such as stress generated by uncertainty and temporary housing conditions [28].

Similarly, other studies have documented that in Indigenous populations with high exposure to structural violence and forced displacement, disruption in child-rearing practices and economic precariousness contribute to the appearance of depressive and affective symptoms in childhood [29–31]. Similarly, the qualitative results of the present study show how in both communities there are concerns about climate change, the impact of contamination due to the armed conflict and the alteration of the relationship with the natural environment.

The relationship between migration, climate change and food security in Latin America and the Caribbean is an interdependent phenomenon that also affects Indigenous populations. High exposure to extreme weather events, such as droughts and floods, has led to the forced displacement of rural communities, reducing their access to sustainable livelihoods and aggravating food insecurity [32]. In this context, it is essential to recognize that Indigenous communities have developed their own forms of early detection of mental health disorders, based on the observation of changes in behavior, spiritual balance and connection with the natural environment [33].

The hypothetical model analysis suggests that climate change is not a direct factor impacting mental health, but rather increases the structural fragility of the community, reducing its adaptive capacity and access to essential resources. In Indigenous communities, the impact of climate change is disproportionate in terms of adaptation, given that these populations depend directly on natural resources for their subsistence. It has been documented that extreme heat, water scarcity and biodiversity degradation affect food security and livelihood opportunities, which in turn influence the emotional stability of the population [32, 34].

Despite the challenges identified, participants highlighted the importance of maintaining traditional practices such as ancestral medicine and environmental education. Indigenous children and youth can be key agents in climate change mitigation, proposing adaptation strategies based on ancestral knowledge [12–14, 30, 32]. In this sense, the Embera Dobidá community has identified environmental recovery and cultural strengthening practices as essential elements to improve their wellbeing [35].

### Limitations

One of the main limitations of the study was the research team’s lack of command of the language, which made it difficult to interpret certain concepts specific to the community. Although local translators were available, some topics were better understood from the direct testimonies of Indigenous children and adolescents. Another limitation was the size of the sample, which, although representative of the community studied, does not allow extrapolations to other Indigenous populations with different cultural characteristics. However, these findings reflect trends observed in multiple Indigenous communities that have experienced forced displacement following the peace process in Colombia.

This study made it possible to incorporate a comparative dimension into the analysis, aimed at exploring the impact of migration on child and adolescent mental health. For security reasons, specific details about the communities were not included, given that they are in a region affected by the armed conflict. Similarly, for security reasons, community leaders did not want to participate in further authorship roles in this article, which is considered a limitation.

The analysis of these populations sought to provide evidence on how displacement and loss of ancestral territory can influence variables such as general health, food security, emotional symptoms and suicidal risk, in the context of a polycrisis that disproportionately affects Indigenous communities. Future research should further explore the relationship between spiritual disharmony and climate anxiety, developing studies that include educational measures and environmental recovery strategies to strengthen wellbeing in vulnerable communities.

In conclusion, the results of this study suggest that mental health problems in Indigenous communities are linked to food insecurity, territorial displacement and environmental degradation. Mental health could be altered in indigenous communities displaced from their traditional territory. The importance of recognizing individual, family and collective forms of healthcare in each Indigenous community is emphasized, ensuring that interventions respect the cultural identity and social conditions of the environment.

The interconnection between food security, climate anxiety and mental health in these Indigenous communities is evidence of the devastating impact of forced displacement and the ecological crisis on their wellbeing. The loss of territory and its transformation affect not only their access to essential resources, but also their identity and emotional balance.

In addition, the interaction between environmental, social and cultural factors should be considered in the formulation of public policies, allowing models of mental healthcare to be comprehensive and culturally relevant. Understanding territory not merely as land, but as a source of identity, food and spiritual balance, is essential in designing culturally grounded mental health interventions.

## References

[1] Pollock NJ Naicker K Loro A Mulay S Colman I. Global Incidence of Suicide Among Indigenous Peoples: A Systematic Review. BMC Med (2018) 16(1):145. 10.1186/s12916-018-1115-6 30122155 PMC6100719

[2] Agudelo-Hernández F Rojas Andrade R Vélez Botero H. Identification of Factors Associated with Suicidal Behavior in Colombian Indigenous Children and Adolescents. Int J Men Health (2024). 10.1080/00207411.2024.2334479

[3] Oliveira AFJ Fialho E Paiva de Araújo JA Naslund JA Barreto ML Patel V The Rising Trends of Self-Harm in Brazil: An Ecological Analysis of Notifications, Hospitalisations, and Mortality between 2011 and 2022. Lancet Reg Health Am (2024) 31:100691. 10.1016/j.lana.2024.100691 38500959 PMC10945432

[4] Leske S Paul E Gibson M Little B Wenitong M Kolves K. Global Systematic Review of the Effects of Suicide Prevention Interventions in Indigenous Peoples. J Epidemiol Community Health (2020) 74(12):1050–5. 10.1136/jech-2019-212368 32788303

[5] Kennedy A Sehgal A Szabo J McGowan K Lindstrom G Roach P Indigenous Strengths-Based Approaches to Healthcare and Health Professions Education - Recognising the Value of Elders' Teachings. Health Educ J (2022) 81(4):423–38. 10.1177/00178969221088921 35531386 PMC9066669

[6] Venugopal J Morton Ninomiya ME Green NT Peach L Linklater R George PN A Scoping Review of Evaluated Indigenous Community-Based Mental Wellness Initiatives. Rural and remote health (2021) 21(1):6203. 10.22605/RRH6203 33730509

[7] Observatorio del Bienestar de la niñez. Suicidio de niños, niñas, adolescentes y jóvenes en Colombia (2018). Available online at: https://www.icbf.gov.co/suicidio-de-ninas-ninos-adolescentes-y-jovenes-en-colombia (Accessed April 10, 2024).

[8] Ministerio de Salud y Protección Social, Asociación de Cabildos Indígenas de CaldasAutoridades Indígenas de Colombia. Manifiestos de las Armonías Espirituales y de Pensamiento de los pueblos indígenas de Colombia. Available online at: https://www.minsalud.gov.co/sites/rid/Lists/BibliotecaDigital/RIDE/VS/PP/ENT/lineamiento-cuidado-armonias-espirituales-pensamiento-pi.pdf (Accessed May 1, 2024).

[9] Azcona Pastor JM Chauca García J. La falacia del exterminio de la población indígena en Hispanoamérica (1492-1898). Cuadernos de Investigación Histórica (2022)(42) 1–12. Available online at: https://www.redalyc.org/articulo.oa?id=691872664005 (Accessed July 16, 2025).

[10] Organización Nacional Indígena de Colombia. Caracterización del Pueblo Embera Dodibá - Gente de rio (2020). Available online at: https://www.onic.org.co/pueblos/1094-embera (Accessed September 8, 2024).

[11] Puertas Rizo M. Suicidio indígena. Trasfondo político de esta situación en un resguardo Embera Dobidá en el Chocó colombiano. 2010-2015. Universidad del Valle (2017). Available online at: https://bibliotecadigital.univalle.edu.co/bitstream/handle/10893/20640/CB%200525706-3489.pdf?sequence=1&isAllowed=y (Accessed October 2, 2024).

[12] McGorry PD Mei C Dalal N Alvarez-Jimenez M Blakemore SJ Browne V The Lancet Psychiatry Commission on Youth Mental Health. Lancet Psychiatry (2024) 11(9):731–74. 10.1016/S2215-0366(24)00163-9 39147461

[13] Benesch T Sergeeva M Wainstock D Miller J. Climate Change, Health, and Human Rights: Calling on States to Address the Health Risks of Climate Change, through the Inter-American Court of Human Rights. Lancet Reg Health Am (2024) 34:100801. 10.1016/j.lana.2024.100801 38881686 PMC11177188

[14] Becht A Spitzer J Grapsas S van de Wetering J Poorthuis A Smeekes A Feeling Anxious and Being Engaged in a Warming World: Climate Anxiety and Adolescents' Pro-Environmental Behavior. J Child Psychol Psychiatry (2024) 65:1270–82. 10.1111/jcpp.14035 38940197

[15] Kotcher J Maibach E Miller J Campbell E Alqodmani L Maiero M Views of Health Professionals on Climate Change and Health: A Multinational Survey Study. Lancet Planet Health (2021) 5(5):e316–e323. 10.1016/S2542-5196(21)00053-X 33838130 PMC8099728

[16] Hatala AR Njeze C Morton D Pearl T Bird-Naytowhow K. Land and Nature as Sources of Health and Resilience Among Indigenous Youth in an Urban Canadian Context: A Photovoice Exploration. BMC Public Health (2020) 20(1):538. 10.1186/s12889-020-08647-z 32312240 PMC7169029

[17] Murphy KM Yoshikawa H Wuermli AJ. Implementation Research for Early Childhood Development Programming in Humanitarian Contexts. Ann N Y Acad Sci (2018) 1419(1):90–101. 10.1111/nyas.13691 29791733

[18] Wilton KS Murphy KM Mahmud A Azam S Habib A Ibrahim I Adapting Reach up and Learn in Crisis and Conflict Settings: An Exploratory Multiple Case Study. Pediatrics (2023) 151(Suppl. 2):e2023060221K. 10.1542/peds.2023-060221K 37125885

[19] Fritz CQ Thomas J Brittan MS Mazzio E Pitkin J Suh C. Referral and Resource Utilization Among Food Insecure Families Identified in a Pediatric Medical Setting. Acad Pediatr (2021) 21(3):446–54. 10.1016/j.acap.2020.11.019 33253935

[20] UNICEF. Estado mundial de la infancia 2019. Niños, alimentos y nutrición, 2019. Available online at: https://www.unicef.org/es/informes/estado-mundial-de-la-infancia-2019 (Accessed June 18, 2022).

[21] Tong A Sainsbury P Craig J. Consolidated Criteria for Reporting Qualitative Research (COREQ): A 32-Item Checklist for Interviews and Focus Groups. Int J Qual Health Care (2007) 19(6):349–57. 10.1093/intqhc/mzm042 17872937

[22] Braun V Clarke V. Guidelines for Reporting Qualitative Research: A Values-Based Approach. Qual Res Psychol (2024) 1–40. 10.1080/14780887.2024.2382244

[23] Agudelo-Hernández F Alvarez ABG. Creation of an Instrument for Pediatric Mental Health in Indigenous People: A Participatory Design. Child Youth Serv Rev (2024) 158:107447. 10.1016/j.childyouth.2024.107447

[24] Alvarez MC Estrada A Montoya EC Melgar-Quiñónez H. Validación de escala de la seguridad alimentaria doméstica en Antioquia, Colombia [Validation of a household food security scale in Antioquia, Colombia]. Salud Publica Mex (2006) 48(6):474–81. 10.1590/s0036-36342006000600005 17326343

[25] Agudelo-Hernández F Guapacha-Montoya M. Mental Health, Solastalgia and Food Insecurity in Colombian Indigenous Communities. EcoHealth (2025). 10.1007/s10393-025-01715-z 40387967

[26] Isee Systems, Inc. Stella Architect Ink version 22. Lebanon (NH): Isee Systems (2022). Available online at: https://www.iseesystems.com/store/products/stella-architect.aspx (Accessed May 12, 2025).

[27] Jomaa L Ouaijan K Nasreddine L. Children in Crisis: Addressing Nutrition amid Conflict and Economic Hardship in Lebanon. Int J Public Health (2025) 70:1608412. 10.3389/ijph.2025.1608412 40123767 PMC11925704

[28] Fliss IN Levi-Shahar M Balmakov Y Mahajni YR Endevelt R Blaychfeld Magnazi M. Detrimental Changes in Individual Health-Promoting Behaviors Among Internally Displaced Israelis. Int J Public Health (2025) 20:70. 10.3389/ijph.2025.1607794 PMC12133527 40470070

[29] Dean G Vitolins MZ Skelton JA Ip EH Lucas CB Brown CL. The Association of Food Insecurity with Mental Health in Preschool-Aged Children and Their Parents. Pediatr Res (2023) 94(1):290–5. 10.1038/s41390-022-02458-1 36599944 PMC10318115

[30] Przeperski J Owusu SA. Children and the Child Welfare System: Problems, Interventions, and Lessons from Around the World. Child Adolesc Soc Work J (2021) 38:127–30. 10.1007/s10560-021-00740-5

[31] Zahidi F Daneshzad E Farahmand MA Noori A Montazer M Ghosn B Associations between Food Insecurity and Common Mental Health Problems: A Systematic Review and Meta-Analyses of Observational Studies. Food Sec (2024) 16:1555–68. 10.1007/s12571-024-01496-3

[32] The Lancet Regional Health-Americas. Navigating the Intricate Links between Migration, Climate Change, and Food Insecurity in Latin America and the Caribbean. Lancet Reg Health Am (2024) 40:100967. 10.1016/j.lana.2024.100967 39763496 PMC11703575

[33] Gallego-Pérez DF Declercq E Saper RB Barnes LL Wardle J. Characterizing Therapeutic Pluralism Policies in Latin America: A Qualitative Content Analysis. J Integr Complement Med (2023) 29(6–7):439–50. 10.1089/jicm.2022.0685 37200459

[34] Kompas T Che TN Grafton RQ. Global Impacts of Heat and Water Stress on Food Production and Severe Food Insecurity. Sci Rep (2024) 14:14398. 10.1038/s41598-024-65274-z 38909134 PMC11193756

[35] Hernández FA Chamorro JM Pastas NM. Spiritual Disharmonies Among the Emberá Dobida: Territorial, Bodily, and Linguistic Suffering. JAYS (2024) 7:27–46. 10.1007/s43151-023-00111-0

